# State Health Officer’s Report

**Published:** 1897-01

**Authors:** 


State Health Officer's Report: The report of the State Health Officer for the years 1895-96 has just been issued from the office of the State printers. It is a pamphlet of some thirty pages, and embraces a report of quarantine and internal sanitation, together with other matters pertaining thereto.

From this report it seems that at Camp Jenner, a camp established at Eagle Pass in the summer of 1895, to care for a large number of negroes who were returning from Mexico on foot, and amongst whom small-pox was rife—there were received in all, 411 refuges; of whom 178 had small-pox, first and last. This camp was turned over to the Marine Hospital service by the State Health Officer of Texas, at the solicitation of the surgeon of that service, Dr. Magruder, because the State had not made provisions for such contingency, and was not prepared to meet it, without greatly exceeding the amount appropriated for the year for the Quarantine Department for all purposes—or—incurring heavy obligations; neither of which was to be thought of.

These negroes were in utter destitution; in rags, and half starved, footsore and weary, and therefore furnished a good subject for small-pox. The mortality was very high, as might have been expected, there being 51 deaths out of 178 cases, or about 29 per cent. This heavy death rate may be attributed in part, no doubt, to the physical condition of the patients—half starved—and exhausted by long travel on foot, run down furthermore by diarrhœa—the result of insufficient and unwholesome food; but there is room to believe that there were other factors, to-wit: These patients were placed in tents, which, at night, and in inclement weather needs must be closed; and several cases were crowded into each tent. When this death rate is contrasted with the average death rate throughout the State, shown further along in this report to have been just ten per cent, there is reason to believe that the fact last stated, the treatment in tents, was detrimental, and contributed to the mortality. The quarantines established at different points throughout the State where small-pox occurred were camps, and as a rule—it being summer time—the patients were treated in the open air. At any rate, the results shown in the Health Officer's report contrast strikingly with that at Camp Jenner.

The cost to the United States government to care for these refugees, from August to October, inclusive, about ninety days, was, according to Surgeon Magruder's report to the bureau of the Marine Hospital Service, $14,123.49, an amount equal to nearly half the yearly appropriation for State quarantine.

The State quarantine officer, Dr. Evans, remained in general charge of the inspection station, having a general supervision over those admitted to, or discharged from quarantine, and notwithstanding this large number of cases of small-pox imported into and quarantined on Texas soil, not a single case occurred outside of the camp.

The total number of cases of small-pox in the State during 1895, was 95, and 16 deaths occurred; in 1896, there were 116, with five deaths,—a total in the two years of 211 cases, with only 21 deaths; exactly ten per cent. At the present time there is not a case of small-pox in the State, to the knowledge of the State health officer, nor a case of any other infectious disease, and not a case has been reported since August, 1896, a most remarkable showing, considering the vast territory over which the State health officer's jurisdiction extends.

A State Chemist and Bacteriologist.—The appropriation of $33,000 for each year, the State health officer found to be sufficient for all purposes, and in the report he asks for the appropriation of a like amount yearly, for the next term; and also asks that "$2000 per annum be appropriated (or so much thereof as may be necessary), to employ an expert in microscopy and chemistry, to make analyses of suspected polluted waters, and bacteriological examinations whenever, in the judgment of the State health officer, such examinations are deemed necessary to protect the public health."

In conclusion, the report of Dr. Swearingen says:

"The great and constant advances in the science of preventive measures against endemic and epidemic diseases imposes this requirement; and, without it, no health department is thoroughly equipped to perform the responsible duties that devolve upon it. Such addition to the staff of the State health department would greatly widen its scope of usefulness."

A copy of the report can be had by application to the State health officer.

